# Recent advances in non-alcoholic fatty liver disease 

**Published:** 2018

**Authors:** Beata Polewiczowska, David Aldulaimi

**Affiliations:** *South Warwickshire Foundation Trust, UK *

 Non-alcoholic fatty liver disease (NAFLD) refers to a wide spectrum of liver damage, ranging from simple steatosis to steatohepatitis, advanced fibrosis and cirrhosis. Non-alcoholic steatohepatitis (NASH) is a more severe form of NAFLD and it can progress to cirrhosis, liver failure and liver cancer. Most patients with NAFLD are asymptomatic. Common findings include elevated liver enzymes and steatosis on liver ultrasound. Liver biopsy is used to distinguish NASH from other forms of liver diseases and can assess the severity of inflammation and grades of liver fibrosis. NAFLD continues to be increasingly recognised as an epidemic across the globe including Africa and the Middle East (1). 

The prevalence of NAFLD is 30-40% in men and 15-20% in women (1). NAFLD is associated with Diabetes Mellitus Type 2 (DM2), obesity and hyperlipidemia. Studies also show that NAFLD increases the risk of chronic kidney disease, osteoporosis, gallstones, colorectal cancers, sleep apnea and polycystic ovary syndrome (1). NALFD has been reported in children and adolescents in Egypt (1). Fatty liver was found in 15.8% of schoolchildren and it has been associated with high triglycerides. Other studies showed a 42.6% prevalence of NAFLD in individuals with DM2 in Africa and Middle East (1). It is estimated that this will continue to increase along with the aging population, obesity, unhealthy diet and high prevalence of DM2 which is a current challenge in Africa and Middle East. In some areas such as Sudan, the prevalence of diabetes is 19.1%. DM2 accounts for over 90% of diabetes cases in Sub-Saharan Africa (1). 

With the rising incidence and prevalence of DM2 and obesity in Africa and Middle East, it is important that accurate estimate of the prevalence of NAFLD is made in order to predict the number of those who will develop advanced liver disease and develop strategies for interventions and treatment of this condition (1). However, this will involve lots of challenges for the healthcare systems in Africa and Middle East such as development of sensitive biochemical markers for diagnosis, high financial cost associated with NAFLD that exceeds most African and Middle East countries, training of health professionals. Liver transplantation would become the most challenging problem for healthcare systems in resource poor countries (1). Finally, education and public health awareness could stop the increasing prevalence of NAFLD in Africa and the Middle East. 

Polycystic ovary syndrome (PCOS) is associated with insulin resistance and metabolic abnormalities. PCOS is present in 15.2% of Iranian women of reproductive age (2). A cross-sectional study examined the prevalence of NAFLD in women diagnosed with PCOS. 75 women with PCOS and 75 healthy controls were involved in this study. Body mass index (BMI), full biochemical profile including total cholesterol, liver functions, glucose and insulin were obtained for each woman enrolled in the study. Ultrasound scan was performed in all subjects to diagnose fatty liver disease. Results showed that triglyceride level, cholesterol, low density lipoprotein, asparate aminotransferase (AST), alkaline phosphatase (ALP) and fasting insulin in women with PCOS were significantly higher than in women without PCOS (2). However, high density lipoprotein and alanine aminotransferase (ALT) were significantly lower in women with PCOS. 53% of women with PCOS had insulin resistance and 29.3% of women without PCOS had insulin resistance. Fatty liver was present in 38.7% of women with PCOS whereas 18.7% of women without PCOS had fatty liver (2). Further analysis showed that higher BMI, ALT, AST and fasting insulin were significant factors associated with fatty liver disease in women with PCOS. This study suggests that prevalence of NAFLD in women with PCOS is higher than other women. It suggests that screening for liver disease should be offered to women diagnosed with PCOS. This may allow for lifestyle changes early that would potentially improve NAFLD outcomes (2). 

Proton Magnetic Resonance Spectroscopy (H-MRS) is a non-invasive method used to measure liver fat whereas transient elastography (FibroScan) allows for non-invasive estimation of fibrosis as stiffness. One prospective study examined the liver fat content using H-MRS in 97 Finnish subjects at baseline and after 11 years follow-up period. Liver stiffness was measured by transient elastography in all subjects (3). Thorough medical history and physical examination were performed in all subjects at baseline visit. Fasting blood samples were taken for measurements of full blood count, renal function, liver functions tests, triglycerides including high and low density lipoprotein, glucose, insulin and free fatty acid concentrations. H-MRS was used for liver fat content measurement in all subjects (3). At follow-up visit, medical history and physical examination were repeated. Fasting blood samples were obtained for the same biochemical parameters as at baseline. H-MRS was repeated at follow-up in order to measure liver fat content. In addition, 92 subjects underwent liver stiffness measurement using transient elastography at a separate visit. 

The results of this study showed that liver fat content decreased slightly by 5% at 11 years. 79% subjects without NAFLD at baseline remained without NAFLD whereas 73% of those with NAFLD at baseline still had NAFLD after 11 years (3). Baseline parameters including obesity, fasting glucose, insulin, triglycerides, liver enzymes and liver fat content predicted NAFLD at 11 years. However, in a multiple logistic regression model liver fat content was the only independent predictor of NAFLD at 11 years (3). At the follow-up visit, 29% of subjects had increased liver stiffness (3). These subjects were significantly more obese, had wider waist circumferences, higher insulin concentrations and blood leukocyte counts at baseline than subjects whose liver stiffness remained below the cut off values (3). Baseline liver fat content was significantly higher in subjects with increased stiffness. 

Probiotics and Prebiotics supplements are known to have desirable effects on glycemic parameters. One study looked at effects of probiotics and prebiotics on adipokines and glycemic parameters in NAFLD patients (4). In this randomized, double blind, placebo controlled trail, 89 patients with NAFLD were divided into three groups to receive either one capsule probiotic and 16 gram prebiotic placebo (probiotic group), or 16 gram prebiotic powder and one probiotic placebo capsule (prebiotic group) or 16 gram prebiotic placebo and one probiotic placebo (control group) for total of 12 weeks. All subjects were instructed to follow weight loss diet and physical activity recommended during the study. Fasting blood samples were taken at baseline and after intervention to measure leptin, adiponectin, insulin and blood sugar (4). The results indicated that serum concentrations of leptin and insulin decreased significantly in the probiotic and prebiotic groups compared with the control group. However, serum concentrations of adiponectin did not change significantly among the three groups. Fasting blood sugar decreased in the prebiotic group. Thus, probiotic and prebiotic supplementation along with lifestyle intervention is much more effective in reducing glycemic parameters and leptin levels compared with the lifestyle intervention alone. 

Free fatty acids and their metabolites are important mediators of lipotoxicity, leading to progression of non-alcoholic fatty liver disease. Studies have shown that hydrogen sulfide plays an important role in hepatic lipotoxicity. It is also known that 3-mercaptopyruvate sulfotransferase (MPST) is a key enzyme that regulates endogenous hydrogen sulfide biosynthesis (5). Free fatty acids contribute to NAFLD via regulation of MPST. Study looking at hepatic MPST expression has been conducted in mice and patients with NAFLD. It showed that hepatic MPST expression was significantly increased in high fat diet mice and patient with NAFLD (5). However, inhibition of MPST significantly reduced hepatic steatosis in high fat diet mice. This study suggests that inhibition of MPST could be a novel therapeutic strategy for treatment of NAFLD. 

Patients with NAFLD and Type 2 diabetes are at high risk for progression of fatty liver disease. A cross-sectional, retrospective study in France has been conducted in order to investigate whether statin and antidiabetic therapies are associated with steatohepatitis and significant fibrosis. In this study 346 patients with Type 2 diabetes and biopsy proven NAFLD have been included. Results indicated that 84% of patients were on antidiabetic therapy and 45% were on statins (6). NASH was present in 57% and 48% of patients had severe fibrosis. NASH was more common in patients treated with metformin or insulin. Severe fibrosis was seen in those treated with sulfonylureas. Statin use was independently and negatively associated with both NASH and severe fibrosis (6). However, insulin and sulfonylureas were independently and positively associated with the presence of NASH and significant fibrosis. This study suggests that statins may have a protective effect against steatohepatitis and significant fibrosis. Wider use of statins should be warranted in high risk diabetic patients with NAFLD. 

Pentoxifiline is known to inhibit lipid peroxidation. It has also anti-inflammatory properties and prevents TNF alpha synthesis which plays an important role in hepatocellular damage, inflammation and fibrogenesis (7). One study observed the effect of Pentoxifylline use on hepatic histological activity and fibrosis of NASH over one year. Total of 35 patients were selected in this single centre, randomized control trial which 25 patients received 1.2 g of pentoxifylline daily along with moderate exercise and dietary advice (treatment group), whereas 10 patients were subject only to lifestyle modification (control group). All subjects had confirmed NAFLD on ultrasound and liver biopsy. All patients were followed for 12 months at 3-month intervals. Liver biopsy was repeated at 12 months in both groups. Results indicated significant improvement in steatosis, lobular inflammation and hepatocyte ballooning in the pentoxifylline treatment group (7). There was some improvement in steatosis in the control group. However, there was no improvement in lobular inflammation or hepatocyte ballooning in the control group. Fibrosis score did not improve in either group (7). This study demonstrated that pentoxifylline can safely and effectively improve histology in patients with NAFLD. 
